# Death of Mrs. Dowd

**Published:** 1902-06

**Authors:** 


### OBITUARY NOTES.

### Death of Mrs. Dowd.

Hepsabeth Marie (nee Davis), wife of Dr. J. Henry Dowd, after a brief illness died Sunday, May 25, 1902, aged 30 years. An operation for appendicitis had been made without avail, and death came as a great shock to the community. Dr. Dowd has the profound sympathy of his professional colleagues and many other friends in the bereavement that has befallen himself and his little daughter, a bright child of four years.

